# Where Do Poor Women in Developing Countries Give Birth? A Multi-Country Analysis of Demographic and Health Survey Data

**DOI:** 10.1371/journal.pone.0017155

**Published:** 2011-02-28

**Authors:** Dominic Montagu, Gavin Yamey, Adam Visconti, April Harding, Joanne Yoong

**Affiliations:** 1 Global Health Group, University of California San Francisco, San Francisco, California, United States of America; 2 Health, Nutrition, and Population Hub, The World Bank, Washington, D.C., United States of America; 3 RAND Corporation, Arlington, Virginia, United States of America; Tulane University, United States of America

## Abstract

**Background:**

In 2008, over 300,000 women died during pregnancy or childbirth, mostly in poor countries. While there are proven interventions to make childbirth safer, there is uncertainty about the best way to deliver these at large scale. In particular, there is currently a debate about whether maternal deaths are more likely to be prevented by delivering effective interventions through scaled up facilities or via community-based services. To inform this debate, we examined delivery location and attendance and the reasons women report for giving birth at home.

**Methodology/Principal Findings:**

We conducted a secondary analysis of maternal delivery data from Demographic and Health Surveys in 48 developing countries from 2003 to the present. We stratified reported delivery locations by wealth quintile for each country and created weighted regional summaries. For sub-Saharan Africa (SSA), where death rates are highest, we conducted a subsample analysis of motivations for giving birth at home. In SSA, South Asia, and Southeast Asia, more than 70% of all births in the lowest two wealth quintiles occurred at home. In SSA, 54.1% of the richest women reported using public facilities compared with only 17.7% of the poorest women. Among home births in SSA, 56% in the poorest quintile were unattended while 41% were attended by a traditional birth attendant (TBA); 40% in the wealthiest quintile were unattended, while 33% were attended by a TBA. Seven per cent of the poorest women reported cost as a reason for not delivering in a facility, while 27% reported lack of access as a reason. The most common reason given by both the poorest and richest women for not delivering in a facility was that it was deemed “not necessary” by a household decision maker. Among the poorest women, “not necessary” was given as a reason by 68% of women whose births were unattended and by 66% of women whose births were attended.

**Conclusions:**

In developing countries, most poor women deliver at home. This suggests that, at least in the near term, efforts to reduce maternal deaths should prioritize community-based interventions aimed at making home births safer.

## Introduction

Reducing the global burden of preventable maternal, neonatal and child deaths is currently a major focus for the global health community. Improving maternal, neonatal, and child health (MNCH) was a key development priority at the June 2010 Group of Eight (G8) Summit and the September 2010 Millennium Development Goals (MDGs) Summit. Although the annual number of maternal deaths worldwide fell from 526 300 in 1980 to 342 900 in 2008 [1], nevertheless only 23 countries are on course to reach Millennium Development Goal 5 (MDG5) [1], the goal of reducing the maternal mortality ratio by 75% by 2015 [2]. The latest estimates from the World Health Organization indicate that each year about 3.7 million children die within the first 28 days and close to 9.7 million children die before their fifth birthday. UNICEF states that progress towards reaching MDG4, the goal of reducing the under-5 mortality rate by two thirds by 2015, is “insufficient” in the Middle East and North Africa, sub-Saharan Africa (SSA), and South Asia [4]. The burden of maternal mortality remains greatest in sub-Saharan Africa [5].

Evidence-based clinical and preventive interventions to reduce this annual death toll are well documented [6]–[9]. However, while it is common to read the phrase “we know what works” in international advocacy efforts for MNCH [10], [11], there is in fact considerable disagreement about how best to deliver these interventions. In particular, there is an ongoing debate about whether more lives could be saved by delivering these interventions via scaled-up health care facilities or by scaling up community-based initiatives [12]–[14].

Pagel and colleagues have argued that the debate on facility-based versus community-based care for improving women's and children's health perpetuates a false dichotomy, and that the “correct balance of approaches crucially depends on the local context” [13]. A better knowledge of the local context would thus help national policy makers to find the right balance between investing in building facilities or in community-based MNCH approaches.

In order to help address this knowledge gap, we set out to examine this “local context” in 48 developing countries. We examined where births are currently occurring (i.e. home versus facility), whether births at home are attended and by whom, and whether place of birth and delivery attendance relates to factors such as women's socioeconomic status and/or the availability and accessibility of health care facilities. If, for example, few women in a particular country give birth in health facilities, even if these are widely available, delivering packages of interventions via facilities is unlikely to be the most effective approach to reaching MDGs 4 and 5 [14].

In order to build a multi-country knowledge base on place of and attendance at birth, we studied 48 countries that have had Demographic and Health Surveys (DHS) conducted since 2003. We then focused specifically on the experiences of Sub-Saharan Africa where maternal mortality rates are highest. We examined place of birth and delivery attendance by income quintile. For a subset of countries where questions were asked about reasons for home birth, we disaggregated the responses first by income (highest and lowest income quintiles) and then by whether the birth was unattended or attended by a medical professional.

## Methods

We conducted a secondary analysis of DHS data on maternal deliveries in 48 developing countries. DHS are nationally-representative household surveys conducted by ICF Macro/MEASURE DHS on behalf of national ministries of health with financial support from the United States Agency for International Development [15]. DHS data have previously been used in cross-country analyses of public and private provision of care [16]–[18].

Over the years, DHS questionnaires have been updated and have subsequently become the next phase of the survey. Our analysis includes only countries with completed Phase 5 or 6 DHS surveys, occurring from 2003 to the present. Earlier survey phases were omitted from our analysis because they lack an integrated measurement of wealth for survey respondents [19], [20]. Table 1 lists the countries included in our analysis and the corresponding survey years.

**Table 1:** DHS countries and survey years.

All DHS Surveys	
Country	Survey Year
**Central Asia/North Africa/Europe**	
Armenia	2005
Azerbaijan	2006
Egypt, Arab Rep.	2008
Jordan	2007
Moldova	2005
Morocco	2004
Turkey	2003
Ukraine	2007
**South Asia**	
Bangladesh	2007
India	2005-06
Nepal	2006
Pakistan	2006-07
**Sub-Saharan Africa**	
Benin	2006
Burkina Faso	2003
Cameroon	2004
Chad	2004
Congo, Rep.	2005
Congo, Dem. Rep.	2007
Ethiopia	2005
Ghana	2008
Guinea	2005
Kenya	2003
Lesotho	2004
Liberia	2007
Madagascar	2003-04
Malawi	2004
Mali	2006
Mozambique	2003
Namibia	2006-07
Niger	2006
Nigeria	2008
Rwanda	2005
Senegal	2005
Sierra Leone	2008
Swaziland	2006-07
Tanzania	2004-05
Uganda	2006
Zambia	2007
Zimbabwe	2005-06
**Latin America + Caribbean**	
Bolivia	2003
Colombia	2005
Dominican Republic	2007
Haiti	2005-06
Honduras	2005-06
Peru	2004-08
**Southeast Asia**	
Cambodia	2005
Indonesia	2007
Philippines	2003

We analyzed individual country datasets to stratify all reported delivery locations by wealth quintile. The delivery locations were based on the answers given to Model Women's Questionnaire question 436 (variable m15): “where did you give birth to [your child]?” [21]. Responses were included for up to five previous births, occurring from 2001 to the survey date. The discrete nominal response variables include: your home, other home, government hospital, government health center, government health post, other public [sector], private hospital/clinic, other private [sector], or other [21]. Sampling weights, calculated and advised by MEASURE DHS for each dataset [22], were applied to individual responses within each country analyzed. Missing responses were omitted from the analysis. Multiple births were counted as separate entries for the analysis.

We then aggregated delivery location into three categories: “public facility” (government hospital, government health center, government health post, and other public [sector] responses); “private facility” (private hospital/clinic, and other private [sector] responses); and “home” (your home and other home responses). “Other” responses were omitted. For each country “public,” “private,” and “home” delivery location results were disaggregated by the wealth quintile variable included within the dataset by manually tabbing each treatment variable by the wealth treatment variable (v190).

Regional summaries of public versus private usage of care by wealth quintile were produced by calculating an average of each country-specific quintile, weighted by total country population as determined by the 2008 World Development Indicators [23]. Regional summaries are aggregations of weighted averages of each country quintile. This aggregate use of relative wealth quintiles may not provide a true regional representation due to differential country-specific wealth levels (for example, a household in the poorest quintile in Kenya may be significantly richer than a household in the poorest quintile in Mali). Unfortunately, the determination of wealth quintiles by MEASURE DHS is based upon country-specific assets, and the same assets are not measured in each country – making true regional wealth quintile breakdowns impossible. To check how representative our aggregate measure is, we conducted an alternative tabulation of country-specific and regional data, using as an absolute income measure the proportion of the population earning less than $1.25 per day as determined by the World Bank. This alternative approach gave similar results to the “regional summary” approach discussed above [24]. Calculations are included in Spreadsheet S1.

For SSA we conducted a subsample analysis of delivery attendance among the aggregated “home” birth respondents using Model Women's Questionnaire question 435 (variable m3a–m3m): “who assisted with the delivery of [your child]?” Possible responses include: doctor, nurse/midwife, auxiliary midwife, traditional birth attendant (TBA), relative/friend, other, or no one. Home births were disaggregated into five categories: “doctor,” “nurse” (nurse/midwife), “other health professional” (auxiliary midwife), “traditional birth attendant,” and “other/none” (relative/friend, other, and no one). For multiple responses, only the highest level professional was included. Using the previously derived country-specific wealth quintile disaggregation, respondents citing home births were then aggregated into the above attendance categories. Similarly, weighted averages by population were calculated to provide a SSA regional estimate.

We analyzed the reasons that women gave for delivering at home (rather than in a facility), by examining responses to the Model Women's Questionnaire question 443 (variable m65a – m65x): “Why didn't you deliver in a health facility?” This survey question was added to Phase 5 surveys in late 2005, and only occurs in datasets for Ghana, Liberia, Namibia, Nigeria, Sierra Leone, Swaziland, Uganda, Zambia, and Zimbabwe. Possible responses were aggregated into three categories: “cost” (cost too much), “access” (facility not open, too far/no transportation, did not know where), and other (don't trust facility/poor quality service, no female provider at facility, husband/family did not allow, not necessary, not customary, other). Using country-specific wealth quintile disaggregation, respondents within the previous “home” delivery classification were aggregated first into skilled and unskilled groups using the previous delivery attendant categories. “Skilled” included those women whose response to question 435 was delivery attendance by a doctor, nurse, other health professional, or TBA. “Unskilled” included those women whose response to question 435 was attendance by “other/none.” Within the “skilled” or “unskilled” subsets, responses were then disaggregated according to the motivations described above for avoiding facilities. Finally, weighted averages by population were calculated across each category to provide a SSA regional estimate.

We downloaded DHS datasets from www.measuredhs.com and analyzed data using Stata 10/SE [25] and Microsoft Excel 2007 [26]. As this study is based on secondary analysis of existing DHS data that are in the public domain, we did not seek approval from an Institutional Review Board.

## Results

### Place of delivery

**Figure 1:**
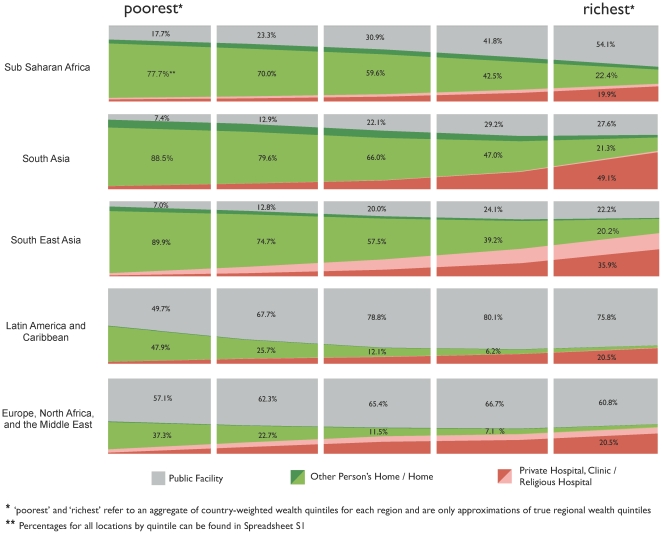
Place of birth by region.

In twenty-three of the 48 countries for which we had data, more than half of births are reported to take place at home (Spreadsheet S1). Home birth is most common among the poor. In SSA, South Asia, and Southeast Asia, 74.7–89.9% of women in the lowest two wealth quintiles reported giving birth at home (Figure 1). In these three regions, even among the wealthiest households 20.2–22.24% of surveyed women reported giving birth at home.

According to survey responses, private hospitals were very rarely used by poor women. In contrast, wealthy women in all regions commonly gave birth in private facilities. In South Asia and South East Asia, 51% and 57%, respectively, of women in the richest quintile reported giving birth in a private or religious facility. In the Latin American and Caribbean region, and in Europe, North Africa, and the Middle East, over 50% of all women in every quintile (except for the poorest quintile in Latin America) reported giving birth in public facilities. In contrast, in the three other regions (SSA, South Asia, and South East Asia), it was much more common for the richest women to use public facilities than the poorest women (e.g. in SSA, 54.1% of the richest women reported using public facilities compared with only 17.7% of the poorest women). In SSA, the poorest women were over three times more likely to report giving birth at home than the richest women (77.7% versus 22.4% respectively). Fewer than 3% of women in SSA as a whole reported giving birth in a religious hospital, though the proportion was higher in some individual countries (e.g. the proportion was 7.8% in the Democratic Republic of Congo, 13.2% in Malawi, and 9.4% in Zimbabwe).

### Home birth attendance

**Figure 2:**
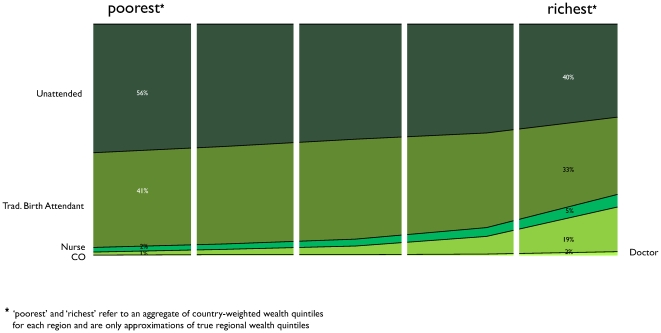
Attended home births in SSA by wealth quintile.

In SSA, although the poorest women were over three times more likely to report giving birth at home than the richest women, both groups of women reported similar rates of non-attendance by a professional. 56% of home births in the poorest quintile were unattended by a trained professional, while 41% of births were attended by a TBA, with the other 3% by a nurse or a doctor. 40% of home births in the wealthiest quintile were reported to be unattended, while 33% were reported to be attended only by a TBA (Figure 2). About one in four home births among the wealthy were attended by a doctor, clinical officer, or nurse. Country-level data on who attended home deliveries are shown in Spreadsheet S2.

### Reasons why women gave birth at home

Within SSA, only nine countries asked women reporting a home birth their reason for not going to a facility (Spreadsheet S2). The responses from these nine countries again showed similarities between wealthy and poor women, but also between those whose births were unattended and those whose birth was attended by a TBA. We grouped responses into three groups—“cost” (i.e. the respondent said the cost was too high); “access” (we used this summary term to group “facility closed,” “too far,” and “did not know where”); and “not necessary” (we used this summary term to group “not necessary,” ‘father did not think necessary,” “‘family did not think necessary,” “husband/family did not allow,” and “not customary.”).

**Figure 3:**
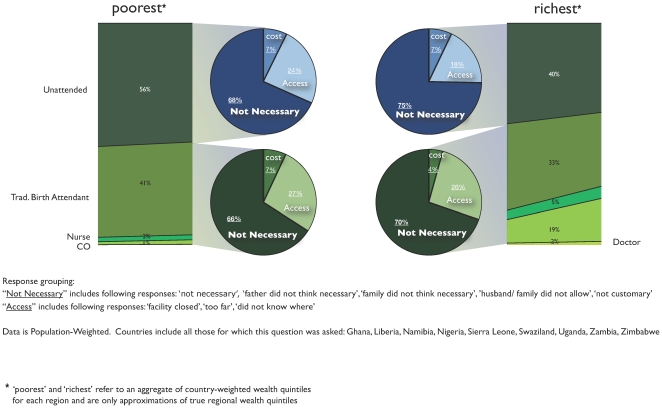
Reasons for not going to a facility for birth, by wealth quintile and attended/non-attended status.

Only 7% of the poorest women reported cost as a deciding reason for not going to a facility for delivery, both among those whose deliveries were unattended and those whose deliveries were attended by a TBA or other clinical provider (Figure 3). Among wealthy women, 7% of women whose deliveries were unattended, and 4% of women whose deliveries were attended by a TBA or other clinical provider, reported cost as the deciding factor. Access was given as a reason more often than cost, by both poor and rich women. Among poor women, access was given as a reason by 24% of women whose births were unattended and by 27% of women whose births were attended. Among wealthy women, access was given as a reason by 18% of women whose births were unattended and by 26% of women whose births were attended.

Among all groups – rich, poor, attended deliveries or unattended deliveries – by far the most common reported reason for not having a facility-based delivery was that it was deemed “not necessary”. Among poor women, “not necessary” was given as a reason by 68% of women whose births were unattended and by 66% of women whose births were attended. Among wealthy women, “not necessary” was given as a reason by 75% of women whose births were unattended and by 70% of women whose births were attended.

## Discussion

Our study had two major findings. First, we found that the richest women in developing countries were much more likely than the poorest to report giving birth in a government facility. Second, we found that a very high proportion of poor women in SSA, South Asia, and South East Asia (about 8 to 9 out of every 10 women) reported giving birth at home. Our study confirms previous research that found that most poor women in the developing world give birth at home [27].

Home births are either unattended or attended. Each category presents different challenges for improving delivery outcomes. In our study, of those women in SSA who reported having a home birth, nearly one-half reported that these births were unattended by any experienced assistant. Nearly 80% reported that they were unattended by a formally trained professional. The large number of unattended home births remains an important barrier to reducing maternal mortality worldwide, particularly for the poor [5].

Our study uses newly available data and updated asset quintile disaggregation to give new insights into *which* women in developing countries are delivering at home and with what level of assistance. This disaggregation by wealth is critical to targeting efforts to reduce maternal mortality and is an important addition to earlier analyses. Our work also provides information on delivery locations, offering insights that may be valuable to programs aimed at achieving MDGs 4 and 5 in the coming five years.

We found that wealthy women in all regions commonly gave birth in private facilities. The role of the private sector in delivering health care in developing countries is much debated. For example, recent studies using DHS data have found an association between private sector participation in health care and better health outcomes or improved health systems performance [16], [17], [27], [28]. In contrast, specifically examining the issue of maternal health and delivery, recent studies have found that strong public sector participation in health care is correlated with better health outcomes [8], [29]–[31]. Our study did not include health outcomes data, so it does not help to resolve the debate.

Several previous studies have examined decisions surrounding the place of birth [29], [32], [33], and concluded that poor availability of facilities is a critical reason why women in the poor world give birth at home [34]. In our study, however, only about a quarter of the poorest women reported lack of facility access (i.e. the facility was too far away, or was closed, or the woman did not know where it was located) as the chief reason for delivering at home. A much high proportion (about two thirds) of the poorest women reported “not necessary” as the chief reason. This motivation for delivering at home is likely to be influenced by social and cultural beliefs, at the household and community levels, related to the value of facility-based care. A similar proportion of the poorest and richest women, and of women whose birth was attended and unattended, reported “not necessary” as a reason for home delivery. Cost was rarely a motivation for delivering at home.

### Limitations

Analysis of DHS data suffers from a number of limitations. Women's reported motivations for delivering at home, which are probably influenced by social, cultural, and economic factors, are likely to be country and region specific, and to change over time. Similarly, women and household decision-makers in different countries are likely to have different views on what constitutes unacceptable cost for facility care, quality, or access (all of which can be motivators for home delivery). But such differences between countries, and such differences over time, could not be detected in our study. In addition, the quantitative survey data do not allow nuanced interpretation of motivation beyond the limited questions asked in nine countries. There has been very little qualitative analysis of the motivation of mothers and other household decision-makers in poor countries for delivering at home.

As noted in the methods section above, the aggregation of country data into regional summaries by wealth quintile introduces known errors through combining wealth quintiles from countries at differing wealth levels. Furthermore, the regional summaries are based on only those countries with DHS surveys fitting our inclusion criteria. In each region a number of countries do not have DHS surveys; our regional aggregates are therefore biased towards countries having a recent DHS survey.

DHS codes for the geographical variables “urban/rural” have different meanings in different countries. As a result we have not included urban/rural analysis in this study; there may be aspects of “wealth” that we are missing as a result. While we did not conduct multivariate analysis of the data in our analysis, we acknowledge that more nuanced findings regarding aspects of care seeking may be elucidated through regression analysis.

We made a decision to use data that best represented all recent births; however this decision over-weights responses given by mothers of multiple births, adding one form of response bias. Recall bias may also have affected our findings. We limited our analysis to the most recent five births, given evidence of recall bias for events beyond five years within DHS surveys. [35] Finally, our regional aggregates are based on weighted total country populations, rather than the total number of married women recently, or potentially, pregnant. While this has reduced the variability of data between countries, we acknowledge that it may have improperly weighted the number of births counted.

We have attempted to address these limitations of our data set by restricting our in-depth analysis to one region (SSA), and to surveys conducted in the recent past – using only DHS rounds 5 and 6 surveys conducted after 2003. This has introduced new limitations; by restricting the interpretation of our findings to SSA, we are unable to draw conclusions about the larger population of developing countries.

While we present summary data in this paper, both the regional and country data are provided in Excel spreadsheet format in Spreadsheets S1 and S2. The verification analysis we did using $1.25 cut-offs for poverty remain an approximation of true wealth. Our verification analysis may be confounded by: (a) the assumptions made by the DHS surveys regarding asset ownership; and (b) the $1.25/day proxy for poverty used by the World Bank, both of which are likely to be highly correlated with each other and with education, marital status, urban/rural residence and other socio-economic factors.

### Meaning and implications of the study

There are two main policy responses to addressing the high numbers of unattended home births among the poor. The first is to scale up facility-based services, and the second is to increase skilled attendance of home births [31]. Between these two choices, the available data provide little evidence for impact from increased supply of facilities [36]; even where facility-based services exist, usage of those facilities remains low [33]. Systematic reviews of MNCH services in developing countries have not been able to provide explanations for why usage rates of facilities remain low [33]. Possible explanations include the impact of cost, access, perceived quality, and cultural preferences for home deliveries [31], [32].

In our study, the proportion of the poorest women reporting home delivery was highest in SSA, which is also the region with the world's highest rates of maternal and child mortality. In this region, where targeting investment is of paramount importance to health outcomes, our analysis of reasons for delivering at home suggests that the motivations for delivering outside of facilities may be primarily social and cultural. Such motivations are not easily addressed through improved access to, or lower cost of, delivery in facilities. Socio-cultural norms shift over time, and long-term investments in facilities may accelerate this shift; meanwhile, cost and access do remain important barriers to the use of facilities for giving birth.

Nevertheless, our findings suggest that, at least in the short term, efforts to reduce maternal and neonatal deaths among the poor should prioritize community-based interventions aimed at making home births safer. Such interventions include those that improve the quality of attended deliveries or increase the rate of delivery attendance. Indeed, systematic reviews have found that training traditional birth attendants can reduce perinatal and neonatal deaths and stillbirths [36].

The global health community is currently focusing its efforts to reduce maternal mortality in developing countries upon two main types of intervention. The first, Emergency Obstetric Care (EmOC) services, have been documented as highly effective at reducing mortality from post-partum hemorrhage, infection, pre-eclampsia, obstructed labor and a range of other causes [35]. The second, community-based interventions with traditional birth attendants (TBAs), better linkages to referral networks, and better informed home-birth partners, has a weaker evidence base [36], although some pilots have had positive results [37]. EmOC services, however, are only of value if the services are used. A review of EmOC services around the world found that while availability of services is poor in some countries, even when these services are available, utilization of EmOC remains low [38], [39]. Low usage is true for both private and public services; interventions to improve the quality of governmental services have proven ineffective at increasing usage [40]. Thus, in the short term at least, in those countries where poor women mostly give birth at home, reducing maternal mortality is likely to require expanding, strengthening, and improving community-based approaches.

### Future research

Our study provides a descriptive analysis of DHS data on place and circumstance of birth. Additional analysis of existing data sets will provide more detailed information for individual countries, both on health-seeking behavior for maternal health generally, and on specific decision making surrounding place of delivery. At the same time, more targeted primary research on this topic is still needed, particularly operations research that can measure the effect of community-based approaches and assess best practices. Two appropriate next steps for health programmers and researchers are: (1) to undertake country and regional multivariate analysis of DHS data and other surveys with information on decision-making surrounding place of birth; and (2) in parallel, to design and test interventions that focus primarily on improving the quality of home based deliveries. The acceptability of home-based interventions to the poor appears likely to be critical for making significant advances towards MGDs 4 and 5 by 2015.

## Supporting Information

Spreadsheet S1 Country-level data on place of birth.
(XLSX)

Spreadsheet S2 Country-level data on who attended home deliveries, and on motivation for delivering at home.
(XLSX)
